# The relationship between interpersonal violence in adulthood and mental health: a longitudinal study based on the Northern Swedish Cohort

**DOI:** 10.1186/s12889-023-15525-x

**Published:** 2023-04-03

**Authors:** Shirin Ziaei, Anne Hammarström

**Affiliations:** 1 Unit of Occupational Medicine, Institute of Environmental Medicine, Karolinska Institutet, Solnavägen 4, 113 65 Stockholm, Stockholm, Sweden; 2 Department of Epidemiology and Global Health, Umeå University, 901 87 Umeå, Sweden

**Keywords:** Interpersonal violence, Mental health, Longitudinal study, Gender, Sweden

## Abstract

**Background:**

Longitudinal studies evaluating the negative effects of exposure to interpersonal violence in the adulthood on the mental health of both women and men are scarce. Using longitudinal data, we evaluated the relationship between the last year experience of violence and functional somatic and depressive symptoms at the ages of 30 and 43 among participants (n = 1006; 483 women and 523 men) in the Northern Swedish Cohort. Further, the relationship between cumulative exposure to violence over a decade and mental health symptoms among participants was evaluated.

**Methods:**

Participants’ experience of interpersonal violence and symptoms of functional somatic and depressive symptoms were evaluated with standard questionnaires at the ages of 30 and 43. General linear models were used to evaluate the relationship between the experience of interpersonal violence and mental health symptoms among the participants. The interactions between gender and violence on functional somatic and depressive symptoms were evaluated separately, and models in which the interaction was significant, were split by gender.

**Results:**

We found that the last year experience of violence at the age of 30 was related to current functional somatic symptoms among all participants and depressive symptoms only among men, (β _Adj_ for the experience of any violence among men: 0.21; CI: 0.12–0.29; Vs. among women: 0.06; CI: -0.04-0.16, p for interaction = 0.02). At the age of 43, last year experience of violence was related to both functional somatic and depressive symptoms in both genders. Finally, a cumulative relationship between the experience of violence over time and mental health symptoms was observed in all participants.

**Conclusions:**

Our findings revealed that while the relationship between the experience of interpersonal violence and mental health symptoms may differ among men and women and with age, the experience of violence can be negatively related to the mental health in both genders.

## Background

Interpersonal violence (IV) is a leading cause of death and the burden of disease globally, which can affect anyone regardless of their gender, ethnicity, or class [1, 2]. According to the World Health Organization, interpersonal violence is defined as “intentional use of physical force or power, threatened or actual, against oneself, another person, or against a group or community, that either result in or has a high likelihood of resulting in injury, death, psychological harm, maldevelopment, or deprivation” [3]. Such violence can occur among family members, intimate partners, friends, acquaintances, and strangers [4]. Due to several negative outcomes such as premature death and lifelong health and social consequences, the associated public health burden and economic impact of interpersonal violence are substantial [5].

The experience of IV is highly gendered. Previous research has shown that men are more likely to experience physical aggression mainly by strangers and the prevalence of violent death is disproportionally higher among men. On the other hand, violence against women is more rooted in inequality and harmful gender norms, with women being more likely to experience sexual abuse or intimate partner violence [6–8]. A great body of literature exists on the link between the experience of IV and several mental health outcomes such as depression, post-traumatic stress disorders and distress among women [9–11], and men [12, 13]. However, little is known about whether the effect of IV on mental health differs between men and women, and if one gender is more vulnerable to the effect of violence compared to the other. There are some studies that suggesting women are more vulnerable to the mental health effects of violence, pointing towards the “feminine vulnerability” hypothesis [6, 14]. On the other hand, there exist other studies arguing that it is not the gender per se but rather women’s higher exposure to violence and revictimization that explains the differences between the genders and suggesting “situational vulnerability” instead of “feminine vulnerability” [7, 15]. In this paper we use the concept of gender, not sex, to emphasise that the differences between men and women are not biologically defined but rather shaped by social and cultural constructions of masculinities and femininities [16].

The experience of IV can occur at every stage of life, and previous research showed interconnection between the experience of violence over the life-course [17]. There exist several pathways through which the experience of violence can affect the mental health of the victims. Violence victimization during life can undermine victims’ sense of self-efficacy and control over life situations [18], diminish their self-identity [19], increase their sense of shame [20], negatively affect their perception of others as a source of threat [21] as well as the perception of self as weak or unworthy [22] and ultimately erode their mental wellbeing over life.

As the nature and experience of violence might be different between men and women, it is valuable to evaluate whether the effect of violence on the mental health differs between men and women. Additionally, previous research constantly shown that the experience of violence is rarely a single incident and that the victims of violence usually experience more than one form of violence and from more than one perpetrator throughout their life-course [23, 24]. Given that IV is a pattern of behaviour and poly-victimization, and re-victimization is common in the experience of violence, it is important to evaluate the cumulative effect of the experience of IV on the mental health of individuals over time.

As in other countries, IV is a social concern in Sweden. Previous research has shown that the experience of violence is a common phenomenon in the country for both women and men. Furthermore, similar to other settings, violence has some gendered features, with men experience more physical violence perpetrated by strangers, while women more often are victims of sexual or psychological violence in their intimate relationships [25, 26].

Although several studies exist on long-term effects of exposure to IV in the adulthood on the mental health of the victims, they have mainly cross-sectional design, retrospectively evaluating the impact of past experience of violence on the current mental health of the participants [27] and largely focused on women [28, 29]. Longitudinal studies evaluating the impacts of IV on the mental health of both genders can be useful in understanding the relationship between the experience of IV and the mental health of the victims over time.

The aim of the paper was to analyse whether the experience of IV (threat of or actual physical violence) during adulthood was related to poor mental health among both genders. We tried addressing the following research questions:

What is the relationship between the last year experience of IV and poor mental health at the age of 30 and 43?

What is the relationship between accumulated exposure to IV during adulthood and poor mental health?

For each of these research questions, we examined possible interactions between violence and gender on mental health symptoms.

## Methods

### Population and study context

The study was embedded in the Northern Swedish Cohort (NoSCo), a 27-year longitudinal follow-up of all pupils who were attending the last year of compulsory school (9th grade) in 1981 in Luleå, a middle-sized industrial town in Northern Sweden. Out of 1083 invited pupils (506 girls, 577 boys), 1080 participated in the study at the baseline at the age of 16. The study participants were followed up during 1983, 1986, 1995, and 2008 at the ages of 18, 21, 30, and 43, respectively, and completed comprehensive questionnaires at each wave of follow-up regarding their socio-economic and social conditions, health status and health behaviour, etc. [30]. The baseline survey was conducted in the classrooms, while the follow-ups were performed in the classmate reunions or via post or telephone. The attrition rate has been extremely low in the cohort with the response rate of 94.3% (of those who were still alive) at the latest follow-up in 2008. The cohort is shown to be comparable to the Swedish population as a whole concerning socio-economic factors, health status and health behaviour. The study has been approved by regional ethics vetting board in Umeå [30].

### Measurements

#### Independent variables

##### Experience of violence at the ages 30 and 43

At the ages of 30 and 43, the participants were asked if they had experienced any threat of or actual physical violence during the last 12 months (Yes = 1/No = 0). Based on their answers to the questions, the following binary variables for the ages of 30 and 43, were created: “experience of any threat of violence” (Yes/No); “experience of any physical violence” (Yes/No) and “experience of any violence (either threat or physical violence)” (Yes/No). Furthermore, an extra variable was created to measure if the participants experienced none, either one or both types of violence (threat of and actual physical violence). In the next step, to capture the cumulative effects of violence at the age of 30 and 43, the following variables were created: “experience of any threat of violence” (none/either at the age of 30 or 43/both at the age of 30 and 43), “experience of any physical violence” (none/either at the age of 30 or 43/both at the age of 30 and 43), and “experience of any violence” (none/either at the age of 30 or 43/both at the age of 30 and 43).

Additionally, respondents were asked about the place where the violence happened with the response options of (at work/at home/somewhere else).

#### Dependent variables

##### Functional somatic symptoms

Functional somatic symptoms (FSS) were operationalised by evaluating cardiopulmonary/autonomic, gastrointestinal, musculoskeletal, and general symptoms through 10 different questions (coded between 0 and 2), which were identical in all waves of data collection. Participants were asked if they had any symptoms of (headache or migraine; stomach-ache other than gastritis or gastric ulcer; nausea; backache, hip pain or sciatica; fatigue; breathlessness; dizziness; overstrain) during the last 12 months with the options of “No = 0”, “Yes light = 1” and “Yes Severe = 2”. Participants’ symptoms of palpitation were measured by the question “How often have you had nervous problems during the past 12 months, with the options of “Never = 0”, “Sometimes = 1”, and “Always = 2”. Additionally, participants’ symptoms of sleeplessness were measured through the question: ‘Have you had sleeping difficulties during the past 12 months’, with the response options of “Never = 0”, “Sometimes = 1” and “Often/Always = 2”. The FFS final score was captured as the mean of 10 items ranging from 0 to 2, with the higher mean indicating more symptoms. The Cronbach’s Alpha for FFS measurement was 0.70, 0.74, and 0.70 for the ages of 16, 30, and 43, respectively, indicating relatively good internal consistency.

##### Depressive symptoms

Depressive symptoms were measured by six questions which were identical in all waves of data collections. The participants were asked if they had the following symptoms during the last 12 months: sleeping problems with four response options ranging from “Never = 0” to “all the time = 3”, poor appetite with three response options “No = 0”, “Yes moderate = 1”and “Yes severe = 2”, general tiredness (coded 0 to 2), feeling down and sad (coded 0 to 3), dejected.

about the future (coded 0 to 3) and concentration difficulties (coded 0 to 2). Response options ranging from 0 to 3 were recoded to 0 to 2 by combining the two middle response options. The final score was captured as the mean of six items ranging from 0 to 2, with the higher mean indicating more depressive symptoms. Cronbach’s Alpha for depressive symptoms measurement was 0.65, 0.67, and 0.76 for the ages of 16, 30, and 43. respectively, indicating relatively good internal consistency.

#### Control variables

Social class has been suggested to be an important predictor of experience of IV as well as mental health [31–33] and thus has been selected as a confounder in this study. The parental occupational class at the age of 16 was selected as the proxy for the social class of the family in which the participants were grew up. The coding was performed based on the Swedish classification system of the Low Income Commission [34]. The classification takes into consideration the educational background needed, job responsibility levels, and specific duties to be performed. Participants with both parents in the working class were classified to have “working-class” background. Those with one parent in the working class were classified as “mixed class” and the ones who had no parents belonging to the working class were classified as having “middle class” background. Considering that the previous mental health symptoms can be predictors of exposure to IV [35] and important indicators of current mental health [36, 37], the models were additionally adjusted for FFS and depressive symptoms at the age of 16.

### Statistical analysis

The normality of the distribution of FFS and depressive symptoms were evaluated and confirmed by Q-Q plats and histogram. Descriptive characteristics of the participants and their experience of violence are presented by frequency and percentages for categorical and mean ± standard deviation (SD) for the continuous variable (FFS and depressive symptoms). General Linear Models (GLM) were used to evaluate the relationships between the experience of violence and mental health symptoms in the total sample. All the models were adjusted for parental occupational class and mental health symptoms at the baseline (age 16). In order to evaluate whether the relationship between IV and mental health of the participants differs by gender, interaction terms were created by multiplying gender and the specific type of violence for each model. Models in which the interaction between gender and violence on functional somatic or depressive symptoms were significant were split by gender and the results were reported separately for men and women. P for the trend was used to evaluate the dose-response relationship between the cumulative exposure to IV during adulthood and FFS and depressive symptoms at the age of 43. The statistical software package IBM SPSS Statistics version 28 (IBM, SPSS, Armonk, NY, USA) was used to perform the analysis.

## Results

Table 1 shows the descriptive statistics of the participants, including background characteristics and distribution of the experience of IV at the ages of 30 and 43 for the total sample and for the women and men separately. Almost one-third of the participants had” working class” background at the age of 16. The mean score of FSS increased while the mean score for depressive symptoms decreased from age 16 to 43, and the women had higher mean scores in FSS and depressive symptoms in all rounds. At the age of 30, 8.7% of the participants (8.2% of women and 9.2% of men) experienced at least one form of IV, either threat of or actual physical violence, i.e. “any interpersonal violence” during the last 12 months. The percentages were slightly higher at the age of 43, with 9.7% of the participants (9.9% of the women and 9.4% of the men) experiencing at least one form of IV. Women experienced IV mainly at the workplace followed by home, while men experienced IV somewhere outside the workplace or at home.

**Table 1:** Descriptive characteristics of the participants and their experience of the violence at the ages of 30 and 43 in total sample and in women and men, separately

Variables	Total Sample	Women	Men
Parental occupational class (age 16); n/N (%)			
None of the parents belonged to occupational class (middle class)	302/1003 (30.1)	147/480 (30.6)	155/523 (29.6)
One parent belonged to occupational class (mixed class)	339/1003 (33.8)	167/480 (34.8)	172/523 (32.9)
Both parents belonged to occupational class (occupational class)	369/1003 (36.1)	166/480 (34.6)	196/523 (37.5)
**Functional Somatic Symptoms Score; mean ± SD (Range)**			
Age 16	0.33 ± 0.25 (0-1.60)	0.37 ± 0.25 (0-1.60)	0.29 ± 0.25 (0-1.20)
Age 30	0.38 ± 0.30 (0-1.63)	0.42 ± 0.30 (0-1.63)	0.33 ± 0.28 (0-1.60)
Age 43	0.42 ± 0.32 (0-1.80)	0.47 ± 0.34 (0-1.80)	0.37 ± 0.29 (0-1.40)
**Depressive symptoms Score; mean ± SD (Range)**			
Age 16	0.48 ± 0.30 (0-1.5)	0.56 ± 0.28 (0-1.50)	0.41 ± 0.29 (0-1.5)
Age 30	0.45 ± 0.32 (0-1.67)	0.51 ± 0.31 (0-1.67)	0.40 ± 0.31 (0-1.67)
Age 43	0.44 ± 0.36 (0-1.83)	0.49 ± 0.37 (0-1.83)	0.39 ± 0.33 (0-1.83)
**Experience of violence at the age of 30; n/N (%)**			
Threat of physical violence	67/1006 (6.7)	30/483 (6.2)	37/523 (7.1)
Physical violence	51/999 (5.1)	21/478 (4.4)	30/521 (5.8)
One type of violence	57/997 (5.7)	27/478 (5.6)	30/519 (5.8)
Both types of violence ^1^	30/997 (3.0)	12/478 (2.5)	18/519 (3.5)
Any violence	87/997 (8.7)	39/478 (8.2)	48/519 (9.2)
Where the violence happened			
- Workplace	29/78 (37.2)	16/36 (44.4)	13/42 (31.0)
- Home	17/78 (21.8)	14/36 (38.9)	3/42 (7.1)
- Somewhere else	32/78 (41.0)	6/36 (16.7)	26/42 (61.9)
**Experience of violence at the age of 43; n/N (%)**			
Threat of physical violence	69/995 (6.9)	34/483 (7.0)	35/512 (6.8)
Physical violence	49/994 (4.9)	23/483 (4.8)	26/511 (5.1)
One type of violence	74/994 (7.4)	39/483 (8.1)	35/511 (6.8)
Both types of violence ^1^	22/994 (2.2)	9/483 (1.9)	13/511 (2.5)
Any violence	96/994 (9.7)	48/483 (9.9)	48/511 (9.4)
Where the violence happened			
- Workplace	38/90 (42.2)	24/45 (53.3)	14/45 (31.1)
- Home	20/90 (22.2)	12/45 (26.7)	8/45 (17.8)
- Somewhere else	32/90 (35.6)	9/45 (20.0)	23/45 (51.1)

1 Threat of and actual physical violence

Table 2 shows the distribution of cumulative experience of IV at the ages of 30 and 43 for the total sample and women and men separately. Among participants, 14% (14.6% of the women and 13.5% of the men) experienced at least one form of IV either at the age of 30 or 43, while 2.2% of the participants (1.7% of the women and 2.7% of the men) experienced either form of IV both at the age of 30 and 43.

**Table 2:** Descriptive statistics of cumulative experience of violence at the age of 30 and 43 in total sample and in women and men separately

Variables	Total sample	Women	Men
Cumulative experience of violence			
Threat of violence; n/N (%)			
Experienced either at 30 or 42	106/962 (11.0)	52/469 (11.1)	54/493 (11.0)
Experienced both at 30 and 42	13/962 (1.4)	5/469 (1.1)	8/493 (1.6)
**Physical violence; n/N (%)**			
Experienced either at 30 or 42	78/956 (8.2)	38/465 (8.2)	40/491 (8.1)
Experienced both at 30 and 42	9/956 (0.9)	3/465 (0.6)	6/491 (1.2)
**Any violence; n/N (%)**			
Experienced either at 30 or 43	134/954 (14.0)	68/465 (14.6)	66/490 (13.5)
Experienced both at 30 and 43	21/954 (2.2)	8/465 (1.7)	12/490 (2.7)

### Experience of interpersonal violence during the last 12 months and current symptoms of mental health

Tables 3 and 4 show the relationships between the experience of different forms of IV during the last 12 months and FFS and depressive symptoms at the age of 30 and 43 in the total sample.

**Table 3:** Relationship between experience of violence during the last 12 months and FSS and depressive symptoms at the age of 30 in total sample. Results from General Linear Models (GLM)

	Functional Somatic Symptoms (FSS) at the age of 30		Depressive symptoms at the age of 30	
Experience of violence at the age of 30	Total sample (n = 997 to 1006)^1^		Total sample (n = 997 to 1006)^1^	
Type of violence	B (95% CI)		B (95% CI)	
	Unadj	Adj^2^	Unadj	Adj^3^
**Experience of threat of violence**				
No experience	Ref	Ref	Ref	Ref
Experienced	0.12** (0.04, 0.19)	0.12** (0.05,0.19)	0.15** (0.08, 0.23)	0.15** (0.07,0.22)
**Experience of physical violence**				
No experience	Ref	Ref	Ref	Ref
Experienced	0.16** (0.08,0.24)	0.16** (0.08, 0.23)	0.18** (0.09.0.26)	0.14** (0.06.0.22)
**Experience of one or both types of violence**				
No experience	Ref	Ref	Ref	Ref
Experience of one type	0.07 (-0.01,0.15)	0.08* (0.00,0.15)	0.10* (0.02,0.19)	0.11** (0.03,0.19)
Experience of both types ^4^	0.21** (0.10,0.31)	0.20** (0.10,0.30)	0.23** (0.11,0.34)	0.18** (0.07,0.29)
**Any violence**				
No experience	Ref	Ref	Ref	Ref
Experienced	0.12** (0.05, 0.18)	0.12** (0.06,0.18)	0.15** (0.08, 0.22)	0.14** (0.07,0.20)

Abbreviations: CI: Confidence Interval, FFS: Functional Somatic Symptoms

1 Total number changed due to missing values in response to the violence experiences

2 Models adjusted for parental occupational class and FFS at the age of 16

3 Models adjusted for parental occupational class and depressive symptoms at the age of 16

4 Threat of and actual physical violence, *p < 0.05, **p < 0.01

**Table 4:** Relationship between experience of violence during the last 12 months and FSS and depressive symptoms at the age of 43 in total sample. Results from General Linear Models (GLM)

	Functional Somatic Symptoms (FSS) at the age of 43		Depressive symptoms at the age of 43	
Experience of violence at the age of 43	Total sample (n = 994 to 995)^1^		Total sample (n = 994 to 995)^1^	
Type of violence	B (95% CI)		B (95% CI)	
	Unadj	Adj^2^	Unadj	Adj^3^
**Experience of threat of violence**				
No experience	Ref	Ref	Ref	Ref
Experienced	0.21** (0.13,0.29)	0.21** (0.13,0.28)	0.28** (0.20,0.37)	0.27** (0.18,0.35)
**Experience of Physical violence**				
No experience	Ref	Ref	Ref	Ref
Experienced	0.33** (0.24,0.42)	0.33** (0.24,0.42)	0.35** (0.25,0.45)	0.34** (0.24,0.44)
**Experience of one or both types of violence**				
No experience	Ref	Ref	Ref	Ref
Experience of one type	0.12** (0.05,0.20)	0.12** (0.04,0.19)	0.17** (0.09,0.26)	0.14** (0.06,0.22)
Experience of both types ^4^	0.50** (0.36,0.63)	0.49** (0.36,0.62)	0.56** (0.42,0.71)	0.56** (0.42,0.71)
**Any violence**				
No experience	Ref	Ref	Ref	Ref
Experienced	0.21** (0.14,0.28)	0.21** (0.14,0.27)	0.26** (0.19,0.34)	0.24** (0.17,0.31)

Abbreviations: CI: Confidence Interval, FFS: Functional Somatic Symptoms

1 Total number changed due to missing values in response to the violence experiences

2 Models adjusted for parental occupational class and FFS at the age of 16

3 Models adjusted for parental occupational class and depressive symptoms at the age of 16

4 Threat of and actual physical violence

**p < 0.01

In the adjusted analysis at the age of 30, experience of the threat of violence, physical violence, and any type of IV were significantly related to higher means in FFS and depressive symptoms among participants. Further, the means in the FFS and depressive symptoms were significantly higher among the participants who experienced both forms of IV (threat of and actual physical violence) (Table 3). There were significant interactions between the gender and threat of violence (p = 01), gender and the experience of one or both types of IV (threat of and actual physical violence) (p = 0.04) and gender and the experience of any IV (p = 0.02) regarding the depressive symptoms at the age 30. Splitting the analysis based on the gender showed that there were significant relationships between the experience of the threat of violence (β _Adj_ 0.24; CI: 0.14–0.34); one (β _Adj_ 0.16; CI: 0.05–0.26) or both types of IV (threat of and actual physical violence) (β _Adj_ 0.29; CI: 0.15–0.42); any IV (β _Adj_ 0.21; CI: 0.12–0.29) and the depressive symptoms among men. The relationship between any of these forms of IV and depressive symptoms was not significant among women (β _Adj_ for the experience of threat of violence: 0.04; CI: -0.04-0.15); (β _Adj_ for the experience of one type of IV :0.06; CI: -0.05-0.18); (β _Adj_ for the experience of both types of IV: 0.04; CI: - 0.13–0.22); (β _Adj_ for the experience of any IV: 0.06; CI: -0.04-0.16) (results not shown in the table).

At the age of 43, experience of the threat of violence, physical violence, and any type of IV were significantly related to higher means in FFS ad depressive symptoms in participants in adjusted models. Like at the age of 30, participants who experienced both forms of IV (threat of and actual physical violence) had higher means of FFS and depressive symptoms (Table 4). No significant interactions between the gender and experience of different types of IV on functional somatic or depressive symptoms were observed in the analysis.

### Cumulative relationship between experience of interpersonal violence at the age of 30 and 43 and mental health symptoms at the age of 43

Finally, Table 5 shows the relationship between the cumulative experience of IV at the age of 30 and 43 and symptoms of FFS and depressive symptoms at the age of 43. A dose-response relationship between the cumulative experience of IV and symptoms of FFS (p for trend = 0.003) and depressive symptoms (p for trend = 0.003) was observed among the participants (Table 5). No significant interactions between the gender and different types of IV on functional somatic or depressive symptoms were observed in the analysis.

**Table 5:** Relationship between the cumulative experience of violence at the age of 30 and 43 and FSS and depressive symptoms at the age of 43 in total sample. Results from General Linear Models (GLM)

	Functional Somatic Symptoms (FSS) at the age of 43		Depressive symptoms at the age of 43	
Cumulative experience of violence at the age of 30 and 43	Total sample (n = 954 to 962)^1^		Total sample (n = 954 to 962)^1^	
Type of violence	B (95% CI)		B (95% CI)	
	Unadj	Adj^2^	Unadj	Adj^3^
**Experience of threat of violence**				
No experience	Ref	Ref	Ref	Ref
Experienced either at 30 or 43	0.12** (0.05,0.18)	0.12** (0.06,0.18)	0.17** (0.10,024)	0.16** (0.10,0.24)
Experienced both at 30 and 43	0.20* (0.02,0.37)	0.19* (0.02,0.36)	0.27** (0.08,0.47)	0.25** (0.06,0.44)
**Experience of Physical violence**				
No experience	Ref	Ref	Ref	Ref
Experienced either at 30 or 43	0.18** (0.11,0.26)	0.18** (0.10,0.25)	0.20** (0.11,0.28)	0.16** (0.08,0.24)
Experienced both at 30 and 43	0.40** (0.19,0.61)	0.39** (0.19,0.59)	0.40** (0.16,0.63)	0.36** (0.13,0.58)
**Any violence**				
No experience	Ref	Ref	Ref	Ref
Experienced either at 30 or 43	0.11** (0.06,0.17)	0.12** (0.06,0.17)	0.14** (0.07.0.20)	0.13** (0.06.0.19)
Experienced both at 30 and 43	0.22** (0.08,0.36)	0.21** (0.07,0.34)	0.25** (0.10,0.43)	0.21** (0.06,0.36)

Abbreviations: CI: Confidence Interval, FFS: Functional Somatic Symptoms

1 Total number changed due to missing values in response to violence experiences

2 Models adjusted for parental occupational class and FFS at the age of 16

3 Models adjusted for parental occupational class and depressive symptoms at the age of 16

*p < 0.05,**p < 0.01

## Discussion

The aim of this study was to evaluate the relationship between the experience of IV (threat of or any actual physical violence), and symptoms of mental health among women and men in the Northern Swedish Cohort.

Results from this study showed that the experience of IV during the last 12 months was related to FFS and depressive symptoms among participants at the age of 30 and 43. We found gender differences in the relationship between the experience of IV and the depressive symptoms at the age of 30, with a significantly higher score in depressive symptoms among men who experienced IV at this age. Additionally, a dose-response relationship between experience of IV at the age of 30 and 43 and mental health symptoms among participants was observed.

In line with previous studies, women in our study, experienced IV mainly at the workplace or at home, while men were exposed to IV somewhere outside [38].

### Experience of violence during the last 12 months and current symptoms of mental health

We found that the experience of IV during the last 12 months was related to current symptoms of mental health among participants. The negative effects of recent experience of IV on the mental health of the victims have been constantly reported [9, 38, 39].

### Gender differences in the relationship between the experience of IV and mental health

In our study, we found gender differences in the relationship between the experience of IV and depressive symptoms at the age of 30, i.e., the experience of any threat of IV, one or both types of IV (threat of and actual physical violence), and any IV at the age of 30 were significantly related to higher means of depressive symptoms among men while no significant relationship between the experience of these forms of IV and depressive symptoms was absorbed among the women at the age of 30. Research on gender-based differences in the negative impacts of experience of IV on mental health are inconclusive. While research has shown that women are more likely to suffer from mental health symptoms associated with the experience of violence [6, 40, 41], there are some studies suggesting that the impacts of IV on mental health outcomes do not vary by gender [12, 15, 42]. In contrast, some studies have shown that the impacts of IV on mental health are more pronounced in men [13, 43]. The observed difference between men and women, particularly at the age of 30, may be attributed to gender- based differences in the experience of violence. Gender determined higher exposure to particular types of violence. Previous research has shown that the experience of sexual violence is more common among women and strongly predicts their mental health, whereas men are more likely to experience physical assault [6]. Global reports have shown that young men are more likely to be victims of severe physical violence such as homicide and serious assault, and that the number of violent deaths is at its highest pick and almost five times higher in men at the age of 15–29 than in women in a similar age group [5]. The focus in this study was the experience of physical violence (either threat or actual experience), which might be more common and severe and thus perhaps more damaging for the men at the age of 30.

### Cumulative relationship between experience of violence at the age of 30 and 43 and mental health symptoms at the age of 43

We found a dose-response relationship between the experience of IV over time and the mental health of the participants. In line with our findings, previous studies that retrospectively evaluated the cumulative experience of violence, shown that the mental health consequences of IV are incrementally worse for the victims who experience multiple forms of violence either at the same time or over life spam [44–46]. While the cross-sectional design of those studies allowed to only demonstrate associations, findings from our study suggested a temporal relationship between the cumulative experience of IV and mental health outcomes. Given the persistent nature of violence and negative effects accompanied by the experience of violence, it is no surprise that the cumulative experience of violence is related to higher mental health symptoms.

### Strengths and limitations

The strength of this study was the longitudinal nature of the study. The cohort had extremely low attrition and very high participation rate in all follow-ups. The measurements of mental health symptoms have been compared with current golden standards of mental health research and have shown reliable internal consistency [47]. However, this study has certain limitations that need to be considered. Underreporting of the experience of violence is common [48, 49]. Using behaviourally specific questions have been shown to reduce the underreporting of violence [50]. In this study, we broadly asked about participants experience of IV rather than specific acts of violence, which might have been the reason for the low prevalence of IV in our sample. However, if underreporting is the case, it means that the relationship between the experience of violence and mental health symptoms of the participants is stronger. In our study, the questions regarding IV were focused on physical violence, either the threat of or the actual experience of physical violence. Although the experience of physical violence has been recognized as a great source of stress and trauma, studies have shown that the other types of violence, such as sexual or emotional violence, are at least as damaging as physical violence, particularly in women due to their higher exposure [41, 46, 51]. We did not have information regarding participants experience of such violence, which might have led to an underestimation of the negative effects of IV on the mental health of participants, especially among women. Additionally, we did not have data on participants experience of adversities and violence during their childhood. It is well known that childhood experience of adversities is a great predictor of experience of violence as well as poor mental health during the adulthood [52] and thus a confounder for this study. However, we have adjusted our analysis for participants’ mental health symptoms the baseline (Age 16), which can partly be a proxy for experience of adversities during childhood [53]. Previous studies have shown that the experience of violence in the early life can negatively affect the educational and economic attainment of the victims in later life [32, 54], make them more susceptible to alcohol and drug abuse [55] and risky behaviours [56] and eventually affect their mental health. Some of these pathways might be similar in the link between the experience of IV and mental health during adulthood and need to be evaluated in further studies. Finally, due to the relatively small proportion of the participants with experience of violence, we could not stratify the sample and compare whether the negative effects of violence were different based on the place that the violence had happened. It is rational to believe that the impacts of violence on the mental health of participants might differ based on where and by whom the abuse had happened. Further research evaluating these factors is warranted.

## Conclusions and policy implications

In conclusion, we have found while the experience of physical IV (either threat of or actual physical violence) negatively affects mental health of the victims, the relationship between the experience of violence and mental health symptoms may differ among men and women and with age. Further, incremental relationship between the cumulative experience of IV over time and the mental health of the victims was observed. Considering the persistent nature of the violence, research on IV should have longitudinal approach and consider not only the recent but also the past experiences of interpersonal violence and multiple exposure to violence in men and women. Similarly, public mental health interventions supporting victims of interpersonal violence can benefit from addressing the experience of violence over the longer period of time in both genders.

## Data Availability

The data is not publicly available because the Swedish Data Protection Act (1998:204) does not permit sensitive data on humans (like in our study) to be freely shared. The datasets are available based on ethical permission from the Regional Ethical board in Umeå, Sweden, from one of the co-authors (Anne Hammarström).

## List of abbreviations

IV: Interpersonal Violence
NoSCo: Northern Swedish Cohort
FSS: Functional Somatic Symptoms
GLM: General Linear Model
